# The acceptability of exercise prehabilitation before cancer surgery among patients, family members and health professionals: a mixed methods evaluation

**DOI:** 10.1007/s00520-024-08574-4

**Published:** 2024-05-31

**Authors:** Emily Smyth, Louise Brennan, Rachel Enright, Mandeep Sekhon, Jane Dickson, Juliette Hussey, Emer Guinan

**Affiliations:** 1 Department of Physiotherapy, Trinity College Dublin, Dublin, Ireland; 2 Trinity St James’s Cancer Institute, Dublin, Ireland; 3 St George’s, University of London, Population Health Research Institute, London, United Kingdom; 4 The Beacon Hospital, Dublin, Ireland; 5 Department of Surgery, Trinity College Dublin, Dublin, Ireland

**Keywords:** Acceptability, Exercise prehabilitation, Preoperative exercise, Exercise oncology

## Abstract

**Purpose:**

Exercise prehabilitation aims to increase preoperative fitness, reduce post-operative complications, and improve health-related quality of life. For prehabilitation to work, access to an effective programme which is acceptable to stakeholders is vital. The aim was to explore acceptability of exercise prehabilitation before cancer surgery among key stakeholders specifically patients, family members and healthcare providers.

**Methods:**

A mixed-methods approach (questionnaire and semi-structured interview) underpinned by the Theoretical Framework of Acceptability was utilised. Composite acceptability score, (summation of acceptability constructs and a single-item overall acceptability construct), and median of each construct was calculated. Correlation analysis between the single-item overall acceptability and each construct was completed. Qualitative data was analysed using deductive and inductive thematic analysis.

**Results:**

244 participants completed the questionnaire and *n*=31 completed interviews. Composite acceptability was comparable between groups (*p*=0.466). Four constructs positively correlated with overall acceptability: affective attitude (*r*=0.453), self-efficacy (*r*=0.399), ethicality (*r*=0.298) and intervention coherence (*r*=0.281). Qualitative data confirmed positive feelings, citing psychological benefits including a sense of control. Participants felt flexible prehabilitation program would be suitable for everyone, identifying barriers and facilitators to reduce burden.

**Conclusion:**

Exercise prehabilitation is highly acceptable to key stakeholders. Despite some burden, it is a worthwhile and effective intervention. Stakeholders understand its purpose, are confident in patients’ ability to participate, and regard it is an important intervention contributing to patients’ psychological and physical wellbeing.

**Implications:**

•Introduction should be comprehensively designed and clearly presented, providing appropriate information and opportunity for questions.

•Programmes should be patient-centred, designed to overcome barriers and address patients’ specific needs and goals.

•Service must be appropriately resourced with a clear referral-pathway.

**Supplementary Information:**

The online version contains supplementary material available at 10.1007/s00520-024-08574-4.

## Introduction

Prehabilitation is a multi-disciplinary intervention focussed on enhancing preoperative physiological and psychological status [1–4]. Exercise is one component of this multi-disciplinary intervention which aims to increase preoperative fitness with the goal of reducing postoperative complications, hospital length of stay, healthcare costs and enhancing health-related quality of life (HR-QL) [4, 5]. Development of exercise prehabilitation services and data on effectiveness continues to emerge. However, due to intervention timing, health sequelae confronted by the clinical populations, and the inherent challenges in setting up new services, implementation is challenging [6]. To support integration into clinical pathways, factors which influence implementation are considered [7, 8].

Acceptability of an intervention is a key factor impacting implementation, evident across multiple implementation frameworks [7, 9, 10]. Acceptability is a complex concept which is poorly described across existing literature. The lack of clear definitions and the inconsistency of acceptability measures used makes comparison of the existing data challenging. Using a standardised definition and outcome measure, such as the Theoretical Framework of Acceptability (TFA), to examine acceptability will significantly enhance comprehension of this crucial area [11]. The TFA defines acceptability as a multifaceted construct which describes how appropriate the person delivering or receiving a healthcare intervention believes it to be, based on anticipated or experienced cognitive and emotional responses [11]. This framework describes acceptability using seven constructs and one over-arching acceptability construct. The seven constructs represent areas which influence acceptability and include affective attitude, burden, perceived effectiveness, ethicality, intervention coherence, opportunity costs, and self-efficacy [11]. Evaluation of acceptability throughout the stages of intervention development may enhance future uptake of complex intervention including exercise prehabilitation [7].

The influence of relevant stakeholders on the successful implementation of a service is critical [7, 9, 12]. Research on the acceptability of exercise prehabilitation in cancer patients is growing [13–16]. However, to date, acceptability across stakeholder groups including healthcare providers and family members has not been established. Different stakeholder groups have different opinions and priorities, and inclusion of all stakeholders in research is vital to maximise impact and understanding. Assessment of acceptability across different stakeholder groups will identify facilitators and barriers, enabling design of more accessible and effective services [7]. Therefore, the primary aim of this study was to examine the acceptability of exercise prehabilitation among key stakeholders relevant to surgical prehabilitation, including patients, their families and healthcare providers (HCPs).

## Materials and methods

This study utilised an exploratory mixed-methods design to gain in-depth understanding of the acceptability of exercise prehabilitation amongst key stakeholders in surgical prehabilitation. Quantitative data was collected through a cross-sectional survey adapted from the Generic TFA Questionnaire [17]. The quantitative component provided context for the semi-structured interviews which were also underpinned by the TFA and aimed to gain deeper understandings of the constructs of acceptability and how they apply to the experiences of stakeholder groups. This study was conducted in in accordance with the Declaration of Helsinki and ethical approval was granted by Trinity College Faculty of Health Sciences Research Committee in June 2021 (Ref:210202) and by the Beacon Hospital Research Ethics Committee in November 2022 (Ref: BEA0197).

### Participants

Patients and relatives of patients (referred to subsequently as ‘family members’ group) who were waiting on or had undergone oncological resection in the last 12 months, and HCPs involved in the surgical cancer care were included. Stakeholders <18 years and non-English speaking were excluded. As this study was exploratory and descriptive in nature, there was no predetermined sample size. Participants were invited to participate through multiple channels. Invitation emails were circulated to professional bodies, Cancer Charities and Community Cancer Support Centres in Ireland. The survey was circulated online through various social media platforms, and paper versions were distributed through gatekeepers at surgical oncology clinics and physiotherapy services at two hospital sites in Ireland. Informed consent was integrated into the opening section of the survey and was a requirement to proceed. The survey concluded with an invitation to participate in a semi-structured interview. Participants provided a second written informed consent prior to the interview.

### Development of data collection tools

Acceptability was measured quantitatively using an adapted version of the Generic TFA Questionnaire focussing on exercise prehabilitation [17]. This cross-sectional survey was devised using the constructs of acceptability contextualised with exercise prehabilitation specific characteristics. Constructs and definitions are provided in Table 1.

**Table 1:** The Theoretical Framework of Acceptability Constructions of Acceptability definitions

Construct	Definition
Overall Acceptability (single-item score)	How acceptable overall is the intervention
Affective attitude	How an individual feels about taking part in an intervention
Burden	The amount of effort required to participate in an intervention
Perceived effectiveness	How effective at achieving its goal is the intervention perceived to be
Ethicality	How well the intervention fits into a person’s individual value system
Intervention coherence	How well the individual understands the intervention and how it works
Opportunity costs	The extent to which the cost of the intervention is worth it for engagement
Self-efficacy	The person’s confidence that they can complete the intervention.
Composite acceptability	The sum of all seven constructs and the single-item overall acceptability

The adapted questionnaire was reviewed by two experienced exercise prehabilitation researchers (EG and JH) and by the TFA developer (MS) for relevance and accurate adaptation of the TFA constructs. The final wording of each question was agreed through consensus. The survey comprised an eight-item Likert scale questionnaire, with seven questions, each reflecting one construct of acceptability and single-item for overall acceptability. Each question was scored out of five, where one represents low acceptability and five represents high acceptability, and a total composite acceptability score (the sum all constructs) of 40. Additionally, demographics including age, surgical timeframes (patient and family group), years of experience (HCPs), experience with exercise prehabilitation and habitual exercise (all stakeholders) were collected. At baseline, participants received an infographic or animation describing the purpose, components, dose, schedule and mode of delivery of exercise prehabilitation in advance of completing the survey [18].

Similarly, an interview schedule was drafted with at least one question per construct of acceptability and reviewed by EG, JH and MS. Amendments were discussed and modified through consensus. The final interview guide consisted of 8 questions (supplementary data i), each reflecting one construct of acceptability in addition to five questions on demographics. Interviews were completed by telephone or videocall and recorded using a digital audio recorder.

### Data analysis

Quantitative data was analysed using IBM SPSS 26. Distribution was assessed visually. Between group differences were analysed using independent T-test and ANOVA with post-Hoc analysis. Correlation analysis was completed using Spearman’s Rank Correlation. Significance was set as *p*<0.05.

Audio files recorded from semi-structured interviews were transcribed verbatim and pseudonymised. Transcripts were imported into NVivo 20 qualitative data analysis management software (QSR International, Melbourne, Australia). Transcripts were coded independently by two reviewers, who coded either 100% (ES) or 50% (LB) of transcripts and a subset (10%) by MS to ensure accurate mapping onto the framework. Following data familiarisation, data was analysed using a hybrid approach (deductive and inductive) thematic analysis process. Firstly, transcripts were deductively coded into seven predetermined themes based on the seven constructs of acceptability. Secondly, data within each deductive theme was analysed using an inductive thematic approach to identify a range of related topics within each TFA based theme.

## Results

### Participant characteristics

Participant demographics are presented in Table 2. Between June 2021 and April 2023, *n*=244 participants completed the questionnaire (*n*=100 (41%) HCPs, *n*=101 (41.4%) patients, *n*=39 (16%) family members, *n*=4 (1.6%) stakeholder group not defined) and *n*=31 participated in semi-structured interviews.

**Table 2:** Participant characteristics

Total Sample		Survey respondents (*n*=244)	Semi-structured interview participants (*n*=31)
*Patients*		*Survey respondents (n=101)*	*Semi-structured interview participants (n=12)*
Age (years)		54.9 (14)	n/a
Cancer Type	Breast	38 (37.6%)	6 (50%)
	Lung	18 (17.8%)	1 (8.3%)
	Colorectal	5 (4.9%)	-
	Uterine	3 (2.9%)	1 (8.3%)
	Gastric	3 (2.9%)	-
	Ovarian	4 (3.9%)	-
	Prostate	4 (3.9%)	1 (8.3%)
	Bladder	1 (0.9%)	-
	Liver	-	1 (8.3%)
	Kidney	-	1 (8.3%)
	Pancreatic	-	1 (8.3%)
	Other	25 (2.47%)	-
Habitual exercise	Inactive	19 (14%)	-
	<60 minutes	64 (45%)	8 (47%)
	60-150 minutes	47 (34%)	9 (52%)
Timeframe around surgery	Waiting on surgery	30 (29.7%)	3 (25%)
	<6 months post-op	35 (34.7%)	3 (25%)
	6-12 months post-op	32 (31.7%)	6 (50%)
Experience with exercise prehabilitation	Yes	22 (21.8%)	1 (6%)
	No	77 (76.2%)	16 (94%)
	Not reported	2 (2%)	-
Preoperative exercise levels	Inactive	12 (11.9%)	5 (41.6%)
	<60 minutes	36 (35.6%)	-
	60-150 minutes	51 (50.5%)	6 (50%)
Patient group: habitual exercise	Inactive	19 (14%)	-
	<60 minutes	64 (45%)	8 (47%)
	60-150 minutes	47 (34%)	9 (52%)
*Family Members *		*Survey respondents (n=39)*	*Semi-structured interview participants (n=5)*
Age (years)		41.2 (15)	n/a
Family members group: relatives cancer type	Breast	10 (25.6%)	-
	Colorectal	6 (15.4%)	-
	Uterine	5 (12.8%)	-
	Gastric	3 (7.6%)	1 (20%)
	Ovarian	3 (7.6%)	-
	Lung	1 (2.5%)	-
	Oesophageal	1 (2.5%)	-
	Prostate	1 (2.5%)	-
	Bladder	-	1 (20%)
	Kidney	-	1 (20%)
	Pancreatic	-	1 (20%)
	Brain	-	1 (20%)
	Other	9 (23.1%)	-
Family members group: relatives’ timeframe around surgery	Waiting on surgery	2 (5%)	1 (20%)
	<6 months post-op	16 (41%)	1 (20%)
	6-12 months post-op	20 (51%)	3 (60%)
Relatives’ exercise prehabilitation	Yes	10 (26%)	1 (20%)
	No	29 (74%)	4 (80%)
Family member group: relatives’ preoperative exercise levels	Inactive	8 (20.5%)	2 (40%)
	<60 minutes	14 (35.9%)	-
	60-150 minutes	16 (41%)	3 (60%)
Family member group: participants current exercise levels	Inactive	6 (15.4%)	2 (40%)
	<60 minutes	8 (20.5%)	-
	60-150 minutes	25 (64.1%)	3 (60%)
*Healthcare Providers*		*Survey respondents (n=100)*	*Semi structured interview participants (n=14)*
Years of experience		10 (12)	21 (12.6)
Occupation	Surgeon	9 (9%)	-
	Anaesthetist	3 (3%)	5 (36%)
	Doctor	25 (25%)	-
	General Practitioner	-	3 (21%)
	Intensive Care Consultant	-	1 (7%)
	Nurse	26 (26%)	-
	Physiotherapist	25 (25%)	5 (36%)
	Dietitian	5 (5%)	-
	Occupational Therapist	2 (2%)	-
	Hospital Management	3 (3%)	-
	Other	2 (2%)	-
Experience with exercise prehabilitation	Yes	37 (37%)	5 (36%)
	No	63 (63%)	9 (64%)
Habitual exercise habits	Inactive	1 (1%%)	-
	<60 minutes	16 (16%)	5 (35%)
	60-150 minutes	84 (83%)	9 (64%)

Data is expressed as frequency (%) or mean (SD), *n/a* not applicable, *post-op* postoperative

### Cross-sectional survey results

**Fig. 1:**
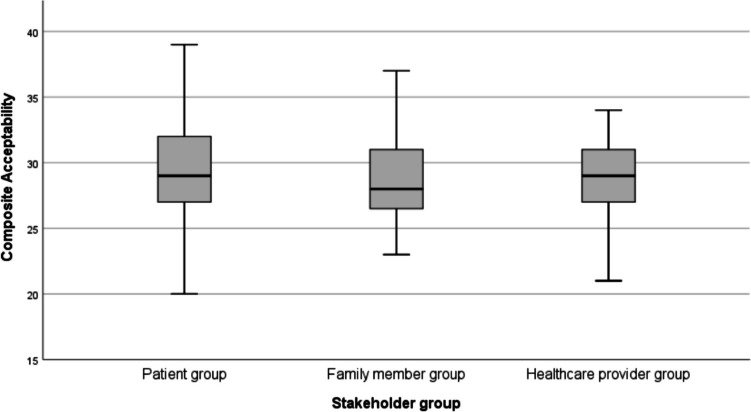
Boxplot of composite acceptability score across stakeholder group

The median (SD) composite acceptability score across all stakeholder groups was 29 (4) out of a maximum of 40 points (29 (4) in the HCPs group, 29 (6) in the patient group and 28 (5) in the family group) (Fig. 1). Composite acceptability scores were comparable between stakeholders (*p*=0.466). Four of the seven constructs of acceptability correlated significantly with the single-item overall acceptability.

In six of the seven constructs, more than 50% of responses were scored at either 4/5 or 5/5, with 5 representing high levels of acceptability (Table 3).

**Table 3:** Median acceptability scores for each construct and correlation analysis between overall acceptability and each construct

Construct	Median (IQR)	% of responses in top two categories (scored 4 or 5 out of 5)	The Spearman’s rank correlation coefficient	p value*
Affective attitude	5 (1)	92%	0.453	<0.001
Self-efficacy	4 (1)	66%	0.399	<0.001
Ethicality	5 (1)	93%	0.298	<0.001
Intervention coherence	5 (1)	89%	0.281	<0.001
Burden	4 (2)	45%	-0.033	0.608
Perceived effectiveness	4 (3)	66%	-0.071	0.275
Opportunity costs	3 (2)	57%	-0.123	0.057

*p-value for correlation analysis of each construct with single-item overall acceptability; *IQR* interquartile range

Sub-analysis of the influence of demographic and clinical characteristics on compositive acceptability scores demonstrated that composite acceptability scores did not vary by habitual physical activity levels in any of the stakeholder groups. Similarly, composite acceptability scores did not differ between patients and family members who had or had no experience with exercise prehabilitation. However composite acceptability scores were significantly higher amongst health professionals who had experience (30(3)) compared to (28(3)) in those without experience of exercise prehabilitation (1.557, 95% CI 0.422-2.692, *p*=0.008). Composite acceptability scores were significantly higher in patients and their family members in the preoperative phase 31(7), compared to 29 (6) less than six months and 28 (4) 6-12 months post-operatively (*p*=0.016). Mean difference in composite acceptability scores pre and post-surgery increased with time from surgery (preoperative and <6months, MD 1.88 (95%CI 0.17-3.16) *p*=0.031, preoperative and 6-12 months MD 2.471 95%CI (0.17-3.16) *p*=0.005). Composite acceptability scores did not correlate with either age (patients/family members) or years of experience (HCPs).

### Semi-structured interview results

Thirty-one participants completed the semi structured interview (*n*=14 HCPs, *n*=12 patients and *n*=5 family members) (Table 2). Results are presented in Table 4.

**Table 4:** Theme & coding structure

Construct	Inductive code	Sample quotes
Affective Attitude	Positive feelings towards prehabilitation Psychological benefits • Improve mood • Reduce stress	*‘ it would have been lovely to have a regime or something that I could work you know give me something you know a targeted goal something I should be working towards if that makes sense’* PT2
Burden	Worthwhile commitment despite burden Minimal effort for physicians to support • Clear referral pathway needed	*‘It certainly is a commitment, but I think for a lot of patients it’s a welcome focus to have at that time point’* FM2
Ethicality	Role in patients’ recovery In line with the health systems values	*‘I think that they will do anything they can to improve the outcome for themselves so high motivation at a time like that’* HCP9
Intervention Coherence	Strong coherence in HCPs • Components involved in prehabilitation • Benefits of participation • Literature on prehabilitation Patients & family required an introduction	*‘I was looking at poster presentations that intervention before major risk surgery like oesophageal cancer reduced time in ICU and reduced mortality and I guess that’s the bottom line’* HCP11
Opportunity Costs	Physiotherapists are under-resourced Patients’ personal commitments may impact ability to prioritise • Work commitments • Family commitments • Large number of appointments	*‘it’s just getting the framework up and running and actually it’s the admin support that’s nearly the hardest bit and then it would be time from physio’* HCP7
Perceived effectiveness	Effective on outcomes Effective at reducing hospital stay	*‘Because all the problems that could arise afterwards your better to spend the money before and to try and prevent rather than deal with it afterwards I think’* PT3
Self-Efficacy	Individualised prehabilitation is appropriate for all patients **Facilitators:** Ability to perform may be enhanced by • A planned and patient focused programme • Clear, educational and empathetic introduction • Accessible to all **Barriers:** Varying levels of ability to perform may be impacted by • Socioeconomic status • Physiological wellbeing • Travel burden	*‘Look, it's going to be difficult for a lot of people if you have cancer, but it really it’s the approaches, the protocols, the benefits. It's how it's presented to the patient it’s the crucial thing’* HCP6

#### Affective attitude

All stakeholders perceived exercise prehabilitation positively. Healthcare providers believed that exercise prehabilitation might enhance patients’ outcomes - ‘*from an anaesthetics perspective I think its brilliant to have your patients in their fittest possible state before they go for their surgery, their outcomes are better’* (HCP4)*.* Physiotherapy participants were particularly passionate about the prospect of exercise prehabilitation, HCP7 reported being ‘*incredibly excited’*. Patients had less experience around exercise prehabilitation and therefore their positive feelings were more modest. However, they generally felt it would be a positive intervention, which could provide support and guidance - ‘*I actually think it’s probably a very good idea’* (PT1). Additionally, participants, particularly HCPs, were aware of the psychological benefits associated with exercise to improve mood and reduce stress at a challenging time- ‘*there’s several benefits to that I think first and foremost that we know there’s a huge body of evidence that says that exercise helps to decrease stress and anxiety’* (HCP10)*.*

#### Burden

A sense of burden was associated with exercise prehabilitation, which may be experienced more by those who are new to exercise *‘I think if it's somebody who's going from zero exercise, it would certainly be more’* (PT4) or those having neoadjuvant treatment. Overall, while burdens exist, they do not necessarily deter individuals from wanting to participate -*’it would have been a lot of effort...but I would have done it’* (PT3). Additionally, HCPs were aware of the burden and financial cost required to establish the service ‘*it’s just getting the framework up and running and actually it’s the admin support that’s nearly the hardest bit*’ (HCP7). Despite the initial workload involved, HCPs felt if funding was received it *‘would be well worth everyone’s while’* to support the delivery of the service. Some participants were concerned that appointments were time-limited and that prehabilitation ‘*may not necessarily be the first thing you discuss with them”*(HCP11), however they felt that once a clear pathway was established, it would reduce the effort involved and the process would easy to support *‘I don’t think it would take that much work, I don’t think the volume of work for us would be too intense’* (HCP9).

#### Ethicality

Exercise prehabilitation may give patients a valuable role in their recovery. At a time when patients are experiencing a loss of control, stakeholders, particularly HCPs, felt that patients would be willing to do whatever it took to help *‘they would do handstands if they thought it would help them get better’* (HCP3). Similarly, patients valued the opportunity to contribute to their recovery journey *‘I would probably have jumped at anything that possibly would have helped me in my quest to get better’* (PT7) and exercise prehabilitation presented this opportunity. Furthermore, HCPs felt prehabilitation had potential to be valuable in the postoperative phase and enhance their ability to provide medical care *‘I think certainly all anaesthetists would be one hundred percent supportive, anything that is going to make our job easier’* (HCP1)*.*

#### Intervention coherence

HCPs had a strong understanding of what prehabilitation involves and the potential benefits. HCPs mentioned literature they had read, suggesting HCPs are actively engaging in the concept of prehabilitation and interested in it *‘obviously it makes the patients fitter and stronger and it certainly improves their short-term outcomes’* (HCP6)*.* Patients were aware of the benefits of exercise but the more formal concept of prehabilitation was new to them*.* There was a desire for introduction and guidance from HCPs to inform and motivate them *‘I would loved to have had a like if you can get to here it will really benefit you or you may not even know that but if there was some way of setting a goal to work towards it might motivate me more if that makes sense* (PT2)*.*

#### Opportunity Costs

Physiotherapists felt that services were under-resourced *‘at this time every employee has a job role to do’* (HCP11)*.* They expressed concern that running exercise prehabilitation programmes without additional staff would have knock-on impacts on other services and physiotherapists’ personal time ‘*…because there was no resources but she said she can't do that going forward she was doing it in the evenings on her own time’* (HCP11)*.* Furthermore, the concern was recognised that initiating the process while still under-resourced would impact the longevity of a prehabilitation programme *‘I think you have to resource something otherwise it is being set up to fail’* (HCP11)*.* Additionally, participants were aware of the significant number of hospital appointments and work or family obligations which may impact patients’ ability to prioritise prehabilitation ‘*how many responsibilities you have got at home, if you have got a heap of kids and nobody to look after* them’ (HCP2)*.* To avoid patients missing out, programmes should be flexible, and prescribed/designed around the patient’s individual needs *‘I can see that actually there can be quite a bit of work around somebody’s lifestyle and thinking about how does this fit into their lifestyle and how likely is it that they are going to comply with this’* (PT1).

#### Perceived effectiveness

Participants felt exercise prehabilitation would increase fitness and in turn may have a positive impact on outcomes - *‘build up that system before it takes the big blow of surgery and hopefully in doing that that would minimise the complications that the patients would have’* (FM2). Overall HCP’s felt that patients who participate in exercise prehabilitation are likely to spend less time in ICU or hospital and that this in turn would have a positive outcome on the economic impact of hospitalisation.

#### Self-efficacy

Stakeholders felt that *‘everybody can do some form’* (HCP1) of exercise prehabilitation. While the level may vary from person to person, everyone should be given the opportunity -*‘I think everybody should be offered some level of exercise that they are being empowered to maximise their possibilities’*(HCP11). Facilitators and barriers which impact ability to participate were identified. Facilitators included provision of a structured, flexible, and individualised prehabilitation programme, which is introduced to patients in a clear and empathetic way. Barriers included travel burden, illness and lower socioeconomic cohort.

## Discussion

There is growing evidence to support the effectiveness of exercise prehabilitation [4]. As this intervention develops, it is vital to consider the uptake and long-term sustainability of the service [7, 9, 10]. This study integrated results from a cross-sectional survey and semi-structured interviews to gather rich information on the acceptability of exercise prehabilitation among key stakeholders in exercise prehabilitation including patients, family members and HCPs. Results indicate that exercise prehabilitation is acceptable to stakeholders; they are positive about exercise before surgery, value its role and feel it is an effective intervention. While exercise prehabilitation is associated with a sense of burden, it was considered a worthwhile commitment, which could be facilitated by enhancing accessibility, flexibility and individualisation of the programme.

Composite acceptability scores were comparable across groups, suggesting that all groups are equally positive regarding exercise prehabilitation. This is an important finding, as patients in this cohort are heavily dependent on support and guidance from their family and HCPs [13–16]. Furthermore, healthcare providers play a particularly vital role as key motivator to patients’ engagement in prehabilitation [14, 16, 19]. Results from the semi-structed interviews similarly emphasised the value of HCPs promoting prehabilitation, particularly the approaches taken by HCPs to disseminate the information. This indicates that patients and their family members not only desire an introduction from HCPs, but also consider the way the topic is addressed as vital to enhancing engagement. These results are consistent with other studies, which found recommendations from HCPS, specifically doctors, were a primary motivator and significantly increased patients’ willingness to participate in exercise prehabiltiation [14, 16, 19].

Results indicate that exercise prehabilitation, like all exercise programmes, is inherently associated with burden [20–25]. The specific burdens identified, such as travel burden, number of hospital appointments and illness are consisted with current literature [20–25]. However, as the results illustrate, burden associated with exercise prehabilitation is complex. Patients and HCPs in the semi-structured interviews expressed concerns that patients who did not regularly exercise at the time of diagnosis may struggle to participate in exercise prehabilitation. However, this is not supported by the quantitative data, where composite acceptability scores are comparable between habitually active and inactive patients and family members. This comparable acceptability may be attributable to the ‘*teachable moment’* concept, often described as an event leading to changes in a person’s health behaviours, such as increased receptiveness to exercise following a cancer diagnosis [26–28]. This willingness to exercise preoperatively, regardless of habitual exercise levels, is evident in a recent systematic review and meta-analysis [29]. Baseline characteristics categorised participants physical fitness as ‘poor or very poor’ compared to normative values, suggesting participants are not habitually active [29]. Despite this, recruitment rates ranged from 38-90.6%, with ‘very poor’ baseline physical fitness for participants in the studies with both the highest and lowest recruitment rates [29]. Upon closer examination of the reasons for declining, in the study with the lowest recruitment rate (38%), travel burden was identified as the primary reason, with no participants declining due to habitual inactivity [30]. These findings support the concept that preoperative habitual exercise levels do not impact willingness to participate in exercise prehabilitation. Furthermore, analysis of the demographic characteristics of participants who expressed this concern revealed that all were HCPs or postoperative patients, and all identified as being habitual exercisers. There is an established link between previous experience with exercise and motivation to participate in survivorship [31, 32]. Therefore, the opinion that inactivity was a barrier to engaging in prehabilitation was largely an assumption, based on current circumstances or observations of other’s (i.e. patients’) behaviour. This may lead them to perceive low levels of habitual activity as a burden for others, despite it not truly being one. This disparity between perceived burden and actual burden may result in a reluctance to address exercise prehabilitation based on assumptions. These results, along with the minimal impact of actual burden on motivation, highlights the importance of addressing exercise prehabilitation with all patients, regardless of preconceptions, allowing the identification of individuals barriers and empowering them to take part.

Prehabilitation brings challenges and considerations for implementation. For patients and family members, pre- or post-surgical status had a clear impact on the acceptability of exercise prehabilitation. Composite acceptability scores were highest in the preoperative group, with levels dropping significantly in the 0-6 months postoperative group and further again in the 6-12 months group. This suggests that patients and family members in the preoperative phase are most motivated and engaged with the idea of exercise prehabilitation compared to other timepoints. This higher acceptability aligns with the ‘*teachable moment’* concept, often described as an event leading to changes in a person’s health behaviours [26–28]. This supports the hypothesis that the preoperative phase may represent an important opportunity not only to participate in exercise, but to educate patients and family members on the role of preoperative and postoperative exercise at a time of highest motivation [4]. This approach is used in smoking cessation, with education and intervention starting following diagnosis with the aim of continuing into survivorship [33, 34]. In the semi-structured interviews, high levels of preoperative motivation to participate in prehabilitation were attributed to a sense of control, at a time where patients felt they had no control. The preoperative phase is associated with fear, isolation and anxiety and participants valued the opportunity for patients to have an active role in the preparation for surgery, a desire consistently identified in pre-treatment oncological cohorts [13, 35–38]. This desire to contribute to preoperative preparation, in addition to the potentially higher capacity to modify health behaviours at this critical time, suggests that the preoperative phase is an opportune time to introduce, educate and motivate patients about exercise.

This study has several strengths and limitations. A strength of this study is the inclusion of family members as their voices are frequently not heard in research, therefore bringing a novel perspective. However, despite a comprehensive recruitment strategy, family members were underrepresented in the overall sample. Additionally, the mean age may limit the generalisability of these results to older adults. Furthermore, the participants in the semi-structured interviews were self-selected, potentially introducing bias as they may have had a greater interest or motivation towards exercise prehabilitation. Additionally, 67% of the patients in this study were in the postoperative phase, while this gives them a unique and valuable insight into the preoperative phase, the majority of the patients did not take part in exercise prehabilitation (20% of all patients), potentially introducing a bias to their perspectives. Furthermore, due to the self-reported nature of the study, data on the type of surgical intervention was not available. We recommend that future work should document these details to enable richer analysis. Finally, while a strength to the study was the use of a theoretical framework to add rigour to the analysis, currently there are no standardised cut-off points for composite acceptability, making quantification of acceptability levels challenging. However, the study was underpinned by a theoretical framework across quantitative and qualitative elements, providing a clear platform for triangulation of results and enhancing the robustness of the results. Furthermore, the publication of multiple protocols utilising this approach will increase the availability of data for comparison, thereby enhancing the ability to compare acceptability levels [39–42].

**Fig. 2:**
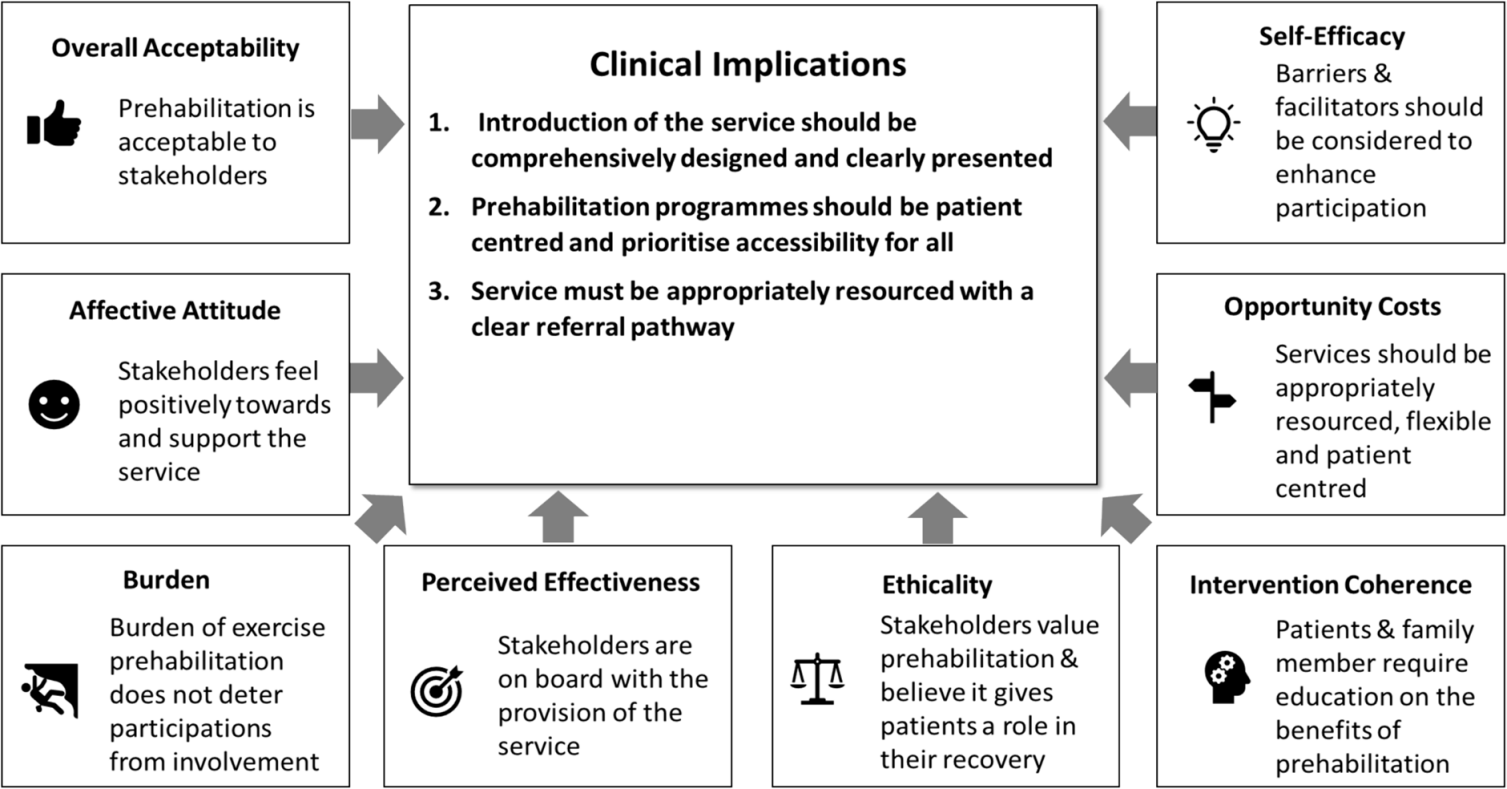
Clinical implications

In conclusion, stakeholders are positive about exercise prehabilitation, and they understand its goal and support the provision of the service. However, consideration should be given to execution of the service to enhance implementation. Therefore, three recommendations have been generated below (please see Fig 2 for additional information).

Introduction of the service should be comprehensively designed and clearly presented. The discussion should be approached in a supportive and accessible manner, discussing potential barriers and empowering patients to participate. The information should include a concise outline of the components of prehabilitation and potential benefits.

Prehabilitation programmes should be patient-centred and prioritise accessibility for all. Programmes should be designed in collaboration with patients, addressing specific needs and goals and enabling them to overcome barriers. Therefore, programmes should be flexible, accommodating of other commitments, and accessible through multiple mediums.

Service must be appropriately resourced with a clear referral process to ensure the longevity of the prehabilitation programme.

### Supplementary information

ESM 1 (DOCX 14 kb)
