# Supplementary material for: The acceptability of exercise prehabilitation before cancer surgery among patients, family members and health professionals: a mixed methods evaluation
Source: Support Care Cancer. 2024 May 31;32(6):399. doi: 10.1007/s00520-024-08574-4 (PMC11142941; doi:10.1007/s00520-024-08574-4)
Supplement: Supplementary file 1 — (DOCX 14 kb) [file 520_2024_8574_MOESM1_ESM.docx]

Semi Structured Interview Guide Sample Questions.

Do you understand what exercise prehabilitation is and how it works?

How do you feel about prehabilitation prior to cancer surgery?

How much of an effort do you feel exercise prehabilitation would be?

Do you feel exercise prehabilitation is of value?

*Prompt*

- What is the value?

Do you feel exercise prehabilitation benefits are worth the cost to of delivering the service?

*Prompt*

- Opportunity costs
- Financial costs

How effective do you feel prehabilitation is?

How confidant do you feel about patient’s ability to participate?
